# Overexpression of V-ATPase B2 attenuates lung injury/fibrosis by stabilizing lysosomal membrane permeabilization and increasing collagen degradation

**DOI:** 10.1038/s12276-022-00776-2

**Published:** 2022-05-27

**Authors:** Jong-Uk Lee, Jisu Hong, Hyesun Shin, Chnag-Beom Ryu, Sung-Woo Park, Sung Hwan Jeong

**Affiliations:** 1 Department of Internal Medicine, Soonchunhyang University Bucheon Hospital, 14584 Gyeonggi-Do, South Korea; 2 Department of Interdisciplinary Program in Biomedical Science Major, Soonchunhyang University Bucheon Hospital, 14584 Gyeonggi-Do, South Korea; 3 Department of Allergy, Pulmonary and Critical Care Medicine, Gachon University, Gil Medical Center, Incheon, South Korea

**Keywords:** Cell adhesion, Experimental models of disease

## Abstract

Excessive oxidative stress causes lysosomal membrane permeabilization (LMP), which leads to cell death. Vacuolar ATPase (V-ATPase) is the enzyme responsible for pumping H^+^ into the cytosol and thus maintaining intracellular pH. Previously, we reported that V-ATPase B2 subunit expression is upregulated in the TiO_2_-exposed lung epithelium. We investigated the role of the lysosomal V-ATPase B2 subunit in oxidative stress-induced alveolar epithelial cell death and in an experimental lung injury/fibrosis model. Overexpression of V-ATPase B2 increased lysosomal pH and lysosomal activities in the cells. In the presence of H_2_O_2_, overexpression of V-ATPase B2 increased survival, and silencing of V-ATPase B2 dramatically increased cell death. Overexpression of V-ATPase B2 diminished H_2_O_2_-triggered LMP, as evidenced by a reduction in acridine orange staining and leakage of cathepsin D from the lysosome to the cytoplasm. In addition, V-ATPase B2-overexpressing macrophages exhibited significantly enhanced uptake and degradation of collagen. V-ATPase B2-overexpressing transgenic mice showed significant inhibition of the bleomycin-induced increases in lung inflammation and fibrosis. We conclude that V-ATPase B2 is critical for maintaining lysosomal activities against excessive oxidative stress by stabilizing LMP. Our findings reveal a previously unknown role of this V-ATPase subunit in a lung injury and fibrosis model.

## Lysoomal vacuolar ATPase B2 prevents oxidative stress-induced cell death and lung injury/fibrosis

An enzyme involved in maintaining the correct pH inside the lysosome, an organelle involved in disposal of cellular waste, also plays a critical role in preventing lung injury. Using human lung cells and mouse models of lung fibrosis, a team led by Sung Woo Park from Soonchunhyang University Bucheon Hospital, South Korea, showed that a overexpression of subunit of the vacuolar ATPase enzyme B2, which pumps protons into cellular compartments to create more acidic environments, helps to boost lysosomal activities that lead to prevent oxidative stress-induced cell death and alleviate experimental lung injury/fibrosis. In addition, V-ATPase B2 overexpressed macrophages increased collagen uptake and degradation activities. The findings point to the vacuolar ATPase, and its B2 subunit in particular, as a promising drug target for future treatments of pulmonary fibrosis.

## Introduction

Reactive oxygen species and their signaling pathways are important factors that regulate many physiological processes. However, oxidative stress caused by an imbalance in the production and clearance of reactive oxygen species is associated with numerous pathological consequences, including necrosis, autophagy, and apoptosis^1^. In idiopathic pulmonary fibrosis (IPF), excessive oxidative stress causes repetitive damage to and eventual death of alveolar epithelial cells, which is the main mechanism in the development of lung fibrosis^2^.

Lysosomes are cytoplasmic membrane-bound acidic organelles that contain numerous hydrolytic enzymes capable of breaking down macromolecules and cell components^3^. They serve as cellular recycling centers for cargo received mainly from autophagy and endocytosis. Normally functioning degradation by lysosomes is essential to maintain cellular homeostasis and enable cell survival in a typical physiological state^4,5^. Lysosomes have long been regarded simply as waste receptacles, although they are now known to play a crucial role in cell death^6,7^. Recent findings have suggested that the involvement of lysosomes in cell death is closely associated with lysosomal membrane permeabilization (LMP)^8,9^. The fate of a cell depends on the extent of lysosomal membrane damage; partial and selective lysosomal leakage results in apoptotic cell death, while a massive rupture of lysosomes and a rapid leak of lysosomal proteases into the cytosol lead to necrosis^8,10^.

Vacuolar H^+^-ATPases (V-ATPases) are a family of ATP-dependent proton pumps that are responsible for acidification of intracellular compartments in eukaryotic cells^11^. Acidification of vacuolar compartments by eukaryotic V-ATPase is essential for several cellular processes, including membrane trafficking^12^, lysosomal proteolysis^13^, elimination of invading microorganisms in phagosomes^14^, and secondary transport of ions and metabolites^15–17^. V-ATPase is a multisubunit complex that contains two domains, namely, an ATP hydrolysis domain (V_1_) and a proton-pumping pore domain (V_0_), the latter of which is required for the translocation of protons across membranes. The V_1_ domain in the mammalian counterpart is composed of eight different subunits (A–H), and the V_0_ domain consists of six different subunits: a, c, c″, d, e, and the accessory subunit Ac45^18^.

Previously, we reported that the protein levels of V-ATPase B2, the B subunit of the ATP hydrolysis domain of V-ATPase, increase in bronchial epithelial cells treated with titanium dioxide (TiO_2_)^19^. Because TiO_2_, a component of particulate matter with a diameter of 10 microns or less, causes upregulation of V-ATPase B2, we speculate that V-ATPase B2 may be involved in the regulation of lysosomal activities against external oxidative stress, although its exact role awaits elucidation.

In the present study, we first investigated the role of the V-ATPase B2 subunit in the oxidative stress state in vitro and in a model of bleomycin (BLM)-induced lung injury and fibrosis. In addition, we studied the mechanisms of its beneficial effects.

## Materials and methods

### Human samples

We obtained human lung tissue samples and bronchoalveolar lavage (BAL) cells from patients with IPF. These samples and our control specimens were all obtained from the Biobank at Soonchunhyang University Bucheon Hospital (Bucheon-si, South Korea). The protocol for this study was approved by the local ethics committee of Soonchunhyang University Bucheon Hospital (SCHBC_IRB_2016–12–024–002).

### Cell culturing

A549 human lung epithelial cells and RAW 264.7 mouse macrophages (ATCC, Manassas, VA, USA) were cultured in Dulbecco’s modified Eagle’s medium or Ham’s F12K medium supplemented with 10% fetal bovine serum (Thermo Fisher Scientific, Rockford, IL, USA), 100 U/mL penicillin, and 100 μg/mL streptomycin (Gibco, Carlsbad, CA, USA). The cells were maintained in a humidified atmosphere with 5% CO_2_ at 37 °C.

### Cloning of the human V-ATPase B2 construct in plasmids

Human V-ATPase B2 cDNA was ligated into the pcDNA 3.1-FLAG-tagged plasmid digested with BamHI and HindIII using T4 ligase (at a molar ratio of insert to vector of 3:1). The ligation mixture was transformed into competent *Escherichia coli* (JM109; Promega, Wisconsin, UK) and plated on lysogeny broth–ampicillin plates. Positive colonies were selected, and the insert in the plasmid was amplified by polymerase chain reaction (PCR) and confirmed by sequencing. The PCR primers were a forward primer, 5′-TACGATACAAGGCTGTTAGAGAG-3′, and a reverse primer, 5′-TAGAAGGCACAGTCG-3′. The PCR program involved predenaturation at 94 °C for 10 min and then 33 cycles of 94 °C for 30 s, 55 °C for 30 s, and 72 °C for 30 s. The PCR products were first separated by electrophoresis in a 1.5% agarose gel to assess the size of the insert, and the positive clones were grown. Then, the recombinant plasmid was purified using a commercial kit (QIAquick Gel Extraction Kit; Venlo, Netherlands), and the insertion sequence was verified by sequencing.

### Generation of a stable human V-ATPase-FLAG-expressing cell line

A549 and RAW 264.7 cells were seeded in a six-well plate and grown in an antibiotic-free medium until they reached 70–80% confluence. The pcDNA V-ATPase B2-FLAG plasmid was transfected into the cells using a reagent (Lipofectamine™ 2000; Invitrogen) following the manufacturer’s instructions. Briefly, solution A contained 250 µL of an improved minimal essential medium (Opti-MEM; Gibco) and 4 µg of plasmid DNA. Solution B contained 250 µL Opti-MEM and 10 µL of Lipofectamine 2000. The two solutions were mixed gently and incubated at room temperature for 20 min. Lipid–DNA complexes were overlaid onto the cells, and the cells were incubated at 37 °C for 48 h in a tissue-culture incubator under 5% CO_2_. Then, the medium was changed to fresh medium containing 500 ng/L G418 antibiotic, and the cells were incubated further; G418-resistant positive clones were isolated after 192 h, and then the individual clones were transferred to the wells of a 24-well plate. The medium was changed again to fresh medium supplemented with antibiotics 1 day later. When the cells had grown to 70% confluence, they were subcultured and expanded by continuous transfer from a six-well plate to a 6 cm plate and then to a 10 cm plate. Overexpression of V-ATPase B2-FLAG was confirmed by immunoblot analysis (Supplementary Fig. 1a). Cells transfected with pcDNA 3.1-FLAG were used as the controls.

### V-ATPase B2 gene silencing in A549 cells using short hairpin RNA lentiviral particles

We used short hairpin RNAs constructed in pLKO.1-puro plasmids (Mission-RNA, Sigma–Aldrich, St. Louis, MO, USA). The pLKO.1-puro plasmid contains a puromycin resistance cassette inserted behind a human phosphoglycerate kinase eukaryotic promoter. For human V-ATPase B2 silencing, we used the clone TRCN000002954, and SHC002 (scramble vector, provided by Sigma–Aldrich) was used as a scramble control. To generate stable V-ATPase B2-knockdown cells, in brief, A549 cells were seeded in a 12-well plate and grown in antibiotic-free medium until they reached 60–70% confluence. The plasmids were added to the cells at a multiplicity of infection of 10 and mixed by gently swirling the plate. We incubated the cell–viral particle mixture with 8 μg/ml polybrene (Sigma–Aldrich) for 24 h at 37 °C and then performed selection with 5 μg/ml puromycin (Invitrogen, Carlsbad, CA, USA) for an additional 72 h. We used immunoblotting to determine the knockdown efficiency (Supplementary Fig. 1b).

### Immunoblotting

Proteins were extracted from lung tissues and cells via incubation in lysis buffer (Thermo Fisher Scientific) with proteinase and phosphatase inhibitor cocktails (Roche Diagnostics) followed by centrifugation. Immunoblotting was performed as described previously^20^. For each experiment, equal amounts of total protein were resolved by 10% sodium dodecyl sulfate-polyacrylamide gel electrophoresis (SDS–PAGE). The proteins were transferred onto polyvinylidene difluoride membranes (Millipore, Billerica, MA, USA) and incubated with specific primary antibodies for 2 h at 37 °C or for 24 h at 4 °C.

After washing several times with phosphate-buffered saline (PBS) containing Tween, the membranes were incubated with an anti-rabbit or anti-mouse immunoglobulin G horseradish peroxidase-conjugated secondary antibody and then subjected to chemiluminescence detection (Bio-Rad, Berkeley, CA, USA) with an imaging system (ChemiDoc™ Touch Imaging System; Bio-Rad).

### Immunohistochemical staining

The lung tissues were dehydrated and embedded in paraffin. For histological examination, sections of samples 4 μm thick and BAL cells on slides were treated with 1.4% H_2_O_2_–methanol for 30 min to block endogenous peroxidase. Then, nonspecific binding was blocked with 1.5% normal serum, and slides were incubated with rabbit anti-V-ATPase B2 polyclonal antibodies (1:200; Santa Cruz Biotechnology Inc., CA, USA). The next day, the sections were incubated with avidin–biotin–peroxidase complex reagents from a kit (ABC Kit; Vector Laboratories, Burlingame, CA, USA). The color reaction was developed by staining with a liquid 3,3′-diaminobenzidine substrate kit (DAB+; Golden Bridge International Inc., Mukilteo, WA, USA). After immunohistochemical staining, the slides were counterstained with Harris’s hematoxylin for 1 min.

### Measurement of V-ATPase activity by measurement of lysosomal pH

We measured the intralysosomal pH of cells overexpressing V-ATPase B2 using seminaphtharhodafluor (SNARF-1) staining and fluorescein isothiocyanate (FITC)-conjugated dextran^21,22^. SNARF-1 is a pH-sensitive fluorophore with biphasic emission. Cells overexpressing V-ATPase B2 were washed, resuspended in a 0.1 M sodium phosphate buffer (pH 7.5), stained with SNARF-1 (final concentration of 125 g/mL; Life Technologies) for 30 min at room temperature, pelleted, washed, and resuspended in fetal bovine serum (FBS)-free RPMI medium. We washed four-well chambered cover glasses (Lab-Tek; Nunc) with FBS-free RPMI and then inoculated SNARF-1-stained cells onto them. These were incubated at 37 °C with 5% CO_2_ for 6 h. Then, the wells were washed and immersed in sodium phosphate buffers of pH 5, 6, 7, or 8. These calibrators and cells were immediately examined with a confocal microscope (DMi 8; Leica, Wetzlar, Germany) using an excitation wavelength of 514 nm and emission wavelength bandpass filters of 555–600 and 640–700 nm. For the dextran method, cells overexpressing V-ATPase B2 grown on coverslips were exposed to 1 mg/mL FITC-conjugated dextran for 24 h at 37 °C in tissue culture medium and washed twice with Dulbecco’s modified Eagle medium. After washing the cells with PBS, the fluorescence emission intensity at 520 nm was measured with excitation at 440 and 490 nm using a spectrofluorometer (VICTOR3^TM^, PerkinElmer Inc, Waltham, USA). The standard curve was generated using 220 μg/mL FITC-dextran in 0.1 M trisaminomethane–HCl or sodium acetate buffers at various pH values.

### Lysosomal activity assay with microscopy and flow cytometry

LysoTracker (Invitrogen, Carlsbad, CA, USA) is a fluorescent acidotropic probe for the labeling and tracking of acidic organelles in live cells. It is highly selective for accumulation in acidic vesicles and is effective for the labeling of live cells^23^. We grew cells in a cell culture dish with H_2_O_2_ (0.5 mM) for 24 h, rinsed them with PBS, and stained them with 500 nM LysoTracker Red (LTR; DND-99; Invitrogen) in serum-free medium for 15 min at 37 °C. Then, the cells were washed with PBS. Lysosomal intensity and size were analyzed using a confocal microscope, and cells with reduced numbers of intact lysosomes (pale cells) were detected by their diminished punctate red fluorescence, as quantified by flow cytometry in the FL3 channel^24^.

### Lysosomal membrane stability assay

Lysosomal membrane stability was assayed using acridine orange (AO) relocation methods. For the AO relocation technique, cells were preloaded with AO (5 μg/mL) for 30 min in complete culture medium, rinsed, and then exposed to H_2_O_2_ (0.5 mM) for 17 h. The increase in green fluorescence due to the release of AO from ruptured lysosomes was monitored under fluorescence microscopy and quantified by flow cytometry in the FL1 channel.

### Apoptosis assays

We exposed cells overexpressing V-ATPase B2 and knockdown cells to H_2_O_2_ (0.5 mM) for 24 h after overnight serum starvation. An annexin V–FITC/propidium iodide detection kit (BD Biosciences Pharmingen, San Diego, CA, USA) was used to determine the proportions of apoptotic and necrotic cells. Aliquots of approximately 1 × 10^6^ cells/mL were washed in PBS, surface-stained, resuspended in binding buffer, incubated with FITC-conjugated annexin V and propidium iodide for 15 min in the dark at room temperature, washed, and resuspended in binding buffer.

### Lysosomal isolation

For subcellular fractionation into cytosolic and membrane/lysosomal fractions, the cells were washed twice with PBS and incubated with twice their volume of MSH buffer (210 mM mannitol, 70 mM sucrose, 20 mM HEPES pH 7.5, 1 mM EDTA, 300 μM Pefabloc, and 100 μM PMSF) for 45 min on ice. Then, they were lysed using a 25-G needle until 50% of the cells were positive for trypan blue. They were centrifuged for 5 min at 350 × *g* to pellet cellular debris and nuclei. The supernatant was centrifuged for 20 min at 16,000 × *g* to obtain the membrane/lysosomal fraction. The resulting supernatant was subjected to 100,000 × *g* centrifugation for 45 min to obtain the cytosol. To obtain a crude membrane/lysosomal fraction for cathepsin Western blotting (containing all organelles except the nuclei), the postnuclear supernatant was directly spun at 100,000 × *g*, and the pellet was resuspended in MSH buffer with 1% Triton. We further assessed the purity of the lysosomal populations by Western blotting for markers of cellular organelles^25^.

### Lysosome-induced protein degradation assay

Purified lysosomes were resuspended in buffer A solution (20 mM HEPES, pH 7.5, 100 mM NaCl, and 0.1 mM dithiothreitol). Prestained bovine serum albumin (BSA) was encapsulated into artificial membranes consisting of phosphatidylserine and phosphatidic acid (molar ratio, 50:50) under acidic buffer conditions (50 mM sodium phosphate, pH 4.7, 50 mM NaCl, and 0.1 mM dithiothreitol) using a reverse evaporation method as described previously^26^. After liposome preparation, the membranes were passed through a protein desalting spin column (Abcam) that was equilibrated with buffer A. To analyze lysosomal protease-induced protein lysis, lysosomal fractions were fused with the BSA-encapsulated membranes, and the fusion efficiencies were evaluated as previously described^27^. The ratio of encapsulated BSA to lysosomes (w/w) for fusion was 2; specifically, 40 µg of BSA in liposomes was mixed with 20 µg of lysosomal proteins, and fusion was started by adding 1 mM CaCl_2_. The reaction was stopped, and lipid components were removed by adding a chloroform–methanol (2:1, v/v) solution. We analyzed the collected protein fractions via 12.5% SDS–PAGE to evaluate BSA degradation.

### Collagen uptake assay

We performed an experiment as described previously^28^. Briefly, macrophages overexpressing V-ATPase B2 or wild-type macrophages were cultured for 60 min on glass inserts and RPMI in 0.1% BSA. FITC-conjugated type I collagen (50 μg/mL) was added, and the cells were incubated for 2 h at 37 °C. After 30 min, the inserts were washed several times to remove unbound and intact collagen, counterstained with 4′,6-diamidino-2-phenylindole, and mounted on slides. The slides were examined via fluorescence microscopy (Axio Observer A1; Zeiss, Oberkochen, Germany); images were obtained at 200× magnification, and a minimum of 500 cells were analyzed for evidence of collagen-binding and ingestion. The investigators were blinded to the experimental conditions when quantifying collagen uptake. The collagen uptake index was the number of ingestions per macrophage, expressed as the percentage of wild-type uptake. The uptake ranged from 3% to 7% for wild-type macrophages. Under visual examination, only cells that had collagen surrounded by a rim of cytoplasm were considered to have uptake. Then, Leica Application Suite X software was used to merge FITC (representing collagen) and phase-contrast (to visualize cytoplasm) images to determine whether collagen was internalized. For collagen to be considered ingested, the maximum fluorescence signal of FITC (representing collagen) and 4′,6-diamidino-2-phenylindole (representing macrophage nuclei) had to be at the same level, and there had to be a clear rim of cytoplasm between the FITC signal and the outside of the cell. Using these criteria, 70% of cells (of 100 counted) that were considered to have uptake by visual analysis also had uptake according to Z-plane analysis. Therefore, the in vitro collagen uptake index represents both ingested (70%) and bound (30%) collagen.

### Collagen degradation assay

Macrophages overexpressing V-ATPase B2 or wild-type (WT) macrophages were cultured in 96-well black plates in triplicate. The cells were plated at a concentration of 50,000 cells per well in PBS with 1 μg/mL FITC-conjugated type I collagen (Invitrogen) in a total volume of 100 μL. FITC-conjugated collagen is designed for enzymatic assays and is supersaturated with FITC. When collagen is cleaved, the fluorescent signal increases. After 2 h, fluorescence was quantified via spectrofluorometry. The fluorescent signal of control wells containing only collagen was subtracted from that of wells containing macrophages and collagen.

### Generation of V-ATPase B2 transgenic mice and establishment of bleomycin-induced lung injury and fibrosis

Inducible human V-ATPase B2 transgenic mice were produced by coinjection of *SP-C-rtTA*-human growth hormone (a gift from Dr. Jeffery Whitsett, Cincinnati Children’s Hospital Medical Center, Cincinnati, OH, USA) and pTRE-Tight-V-ATPase B2-FLAG into C57BL/6 blastocysts (Orient Bio Inc., Charles River Technology, Seongnam, South Korea) as described previously^29^. For cloning of pTRE-Tight-V-ATPase B2-FLAG, human V-ATPase B2 cDNA was inserted into pcDNA 3.1-FLAG, and then a V-ATPase B2-FLAG construct was subcloned into a pTRE-Tight vector (Supplementary Fig. 2a). The Committee on Animal Research at Soonchunhyang University Hospital approved the use of mice for these experiments (SCHBC_Animal_201209). The transgenic mice were given drinking water containing doxycycline (50 mg/mL; Sigma–Aldrich) to induce transgene expression. On Day 0, they received BLM (3 mg/kg) in endotoxin-free water in a total volume of 100 µL by intratracheal delivery. Males (6–8 weeks old) were randomly assigned to two groups (*n* = 8/group). One received drinking water containing doxycycline (50 mg/mL) beginning on Day 0 and continuing until sacrifice on Day 14. The other group received endotoxin-free water containing no doxycycline until sacrifice. As a control, V-ATPase B2 transgenic mice were treated with PBS (100 µL, intratracheally) on Day 0 and housed with or without doxycycline-containing water (50 mg/mL) until sacrifice. At the end of the experimental period, BAL was performed as described previously^30^.

### Statistical analyses

The data were analyzed using SPSS (ver. 20.0; SPSS, Inc., Chicago, IL, USA). All data are expressed as the mean ± standard error of the mean. The data were analyzed using the Kruskal–Wallis test followed by the Mann–Whitney *U*-test (with Bonferroni correction for intergroup comparisons). Significance was defined by a *p* value < 0.05.

## Results

### V-ATPase B2 expression is increased in the lungs of patients with IPF and in the lungs of mice exposed to bleomycin

**Fig. 1:**
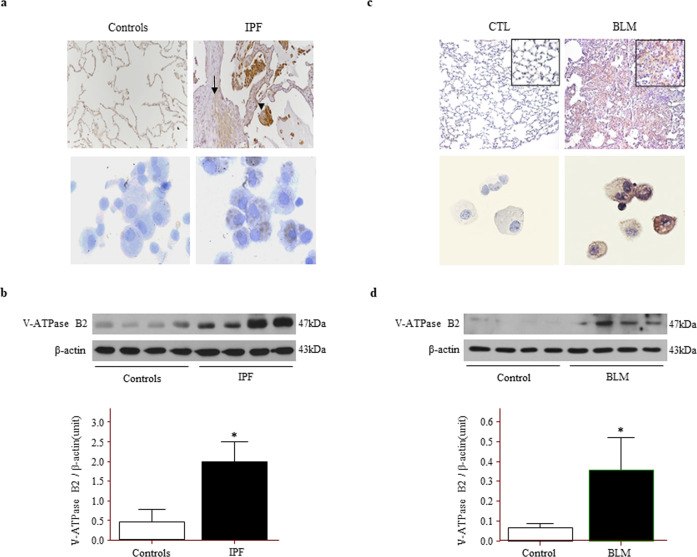
Vacuolar ATPase (V-ATPase) B2 expression is increased in the lungs of patients with IPF and mice treated with BLM. **a** Immunohistochemical staining of V-ATPase B2 in the lungs and bronchoalveolar lavage (BAL) cells of control subjects compared to patients with IPF. Magnification: lungs 100× and BAL cells 400×. The arrows indicate fibroblastic tissue, and the arrowheads indicate pneumocytes. **b** Lung V-ATPase B2 expression measured by immunoblotting and densitometric analysis (*n* = 4 per group). *p < 0.05 versus controls. Lung tissue was obtained from patients with IPF and control subjects who underwent thoracic surgery. **c** Immunohistochemical staining of V-ATPase B2 in mouse lungs and BAL cells. Magnifications: main 100×, inset 1000×, BAL cells 400×. **d** Lung V-ATPase B2 expression measured by immunoblotting and densitometric analysis (*n* = 4 per group). *p < 0.05 versus control. An anti-V-ATPase B2 antibody was used to immunoblot equal quantities of proteins that were obtained from lung lysates. The blots were stripped and re-probed with an anti-β-actin antibody.

V-ATPase B2 was strongly expressed in fibrotic areas in lung tissues and in macrophages in BAL fluid in IPF (Fig. 1a). The protein levels of V-ATPase B2 in lungs measured by Western blotting were significantly higher in the IPF group than in the control group (Fig. 1b). We evaluated whether BLM, a strong inducer of fibrosis due to oxidative stress, enhanced V-ATPase B2 expression in the lungs of mice. Intratracheal treatment with BLM significantly increased V-ATPase B2 expression in C57BL/6 mice, primarily in damaged fibrotic areas and alveolar macrophages (Fig. 1c, d). These results demonstrated that the levels of V-ATPase B2 were mainly increased in the fibrotic areas and alveolar macrophages in patients with IPF and in BLM-exposed lungs.

### More acidic lysosomal pH and increased activity in macrophages overexpressing V-ATPase B2

**Fig. 2:**
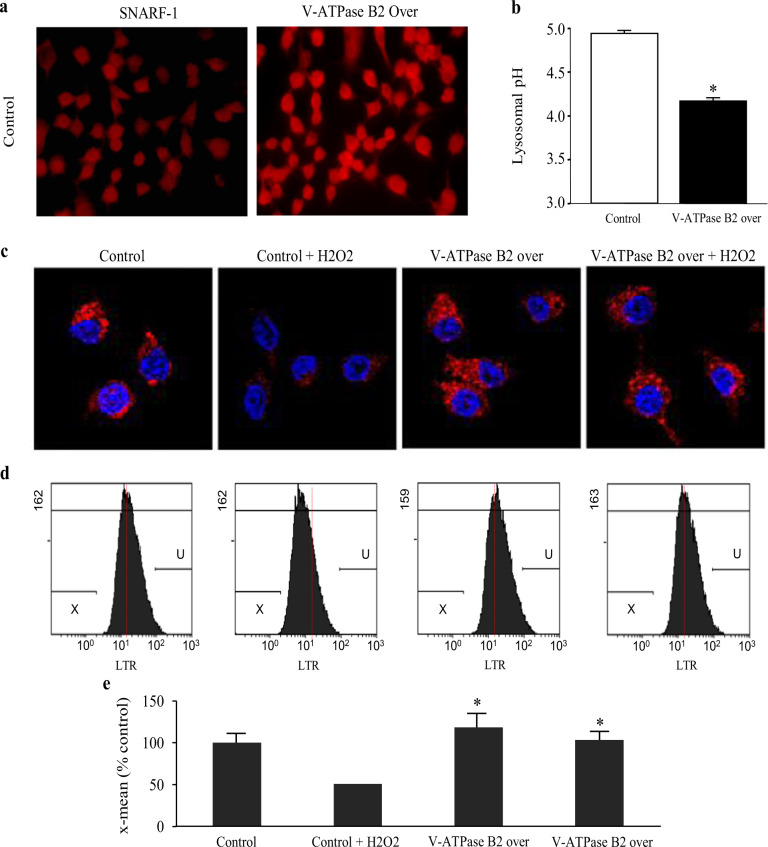
Measurement of intracellular pH and lysosomal activities in V-ATPase B2-overexpressing macrophages. **a** Confocal microscopic image of intracellular pH in living cells using SNARF-1, a ratiometric pH-indicating fluorescent probe. **b** Lysosomal pH measured using fluorescein isothiocyanate-conjugated dextran. The data are expressed as the mean ± standard error of the mean (*n* = 4). *p < 0.05 versus the control groups. **c** Fluorescence intensity of intracellular acidic organelles measured using a LysoTracker probe. LysoTracker is a marker to measure the activity of lysosomes, which are shown in red. **d** Flow cytometry data and **e**. quantitation of the red fluorescence of LTR (a lysosomotropic fluorescent dye) in the cells. The data are expressed as the mean ± standard error of the mean (*n* = 4). *p < 0.05 versus the control or H_2_O_2_-treated control.

Lysosomes must maintain an acidic luminal pH to activate hydrolytic enzymes and degrade internalized macromolecules^31^. We measured intralysosomal pH in B2-overexpressing macrophages using two methods. Confocal imaging of the pH in living cells using SNARF-1, a ratiometric pH-indicating fluorescent probe, showed that B2-overexpressing cells exhibited more intense red than controls, indicating a more acidic pH (Fig. 2a). Then, we measured lysosomal pH using FITC-conjugated dextran and found that it was significantly lower in cells overexpressing V-ATPase B2 than in controls (Fig. 2b).

Lysotracker (LTR) is a lysosomotropic fluorescent dye that accumulates within intact lysosomes, yielding red fluorescence. Rupture of the lysosomal membrane leads to leakage of the probe into the cytosol, resulting in the development of cells that appear dim (pale) under fluorescence microscopy (Fig. 2c), which can be quantified by flow cytometry (Fig. 2d, e). As shown in Fig. 2, exposure of macrophages to H_2_O_2_ for 24 h promoted the appearance of pale cells; however, V-ATPase B2 overexpression maintained the integrity of lysosomal membranes against H_2_O_2_ exposure. This result demonstrates that in the basal state, lysosomal activity, which is reflected by acidic intracellular pH, is increased in cells overexpressing V-ATPase B2 and that V-ATPase B2 maintains lysosomal activity against H_2_O_2_ exposure.

### Lysosomal degradation activity is activated in macrophages overexpressing V-ATPase B2

**Fig. 3:**
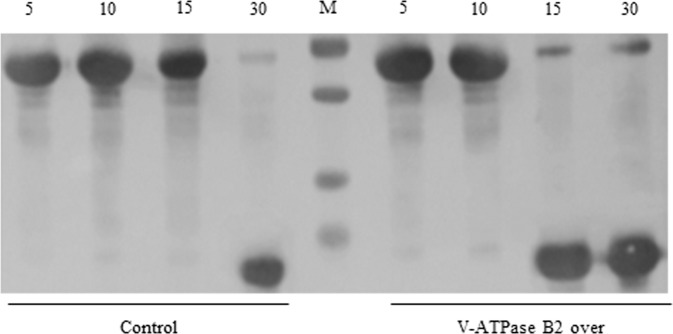
Lysosomal degradation is activated in V-ATPase B2-overexpressing cells. After isolation of the lysosomal fraction from control and RAW 264.7 cells overexpressing V-ATPase B2, 60 µg of bovine serum albumin (BSA) in liposomes was mixed with 30 µg of lysosomal proteins, and fusion was performed by adding CaCl_2_ and incubating for the indicated time periods. The collected protein fractions were analyzed by 12.5% sodium dodecyl sulfate-polyacrylamide gel electrophoresis (SDS–PAGE) to evaluate BSA degradation.

To investigate lysosomal protease-induced protein degradation activity in cells overexpressing V-ATPase B2, lysosomal protease activity was determined using BSA-encapsulated membranes. Liposomal BSA was mixed with lysosomal proteins and then incubated at 30 °C for fusion and simultaneous substrate lysis. SDS–PAGE analysis showed that BSA degradation activity was stronger in cells overexpressing V-ATPase B2 than in control cells, suggesting that overexpression of V-ATPase B2 strengthens lysosomal protease activity (Fig. 3).

### Effect of overexpression and knockdown of V-ATPase B2 on H_2_O_2_-induced cell apoptosis and necrosis

**Fig. 4:**
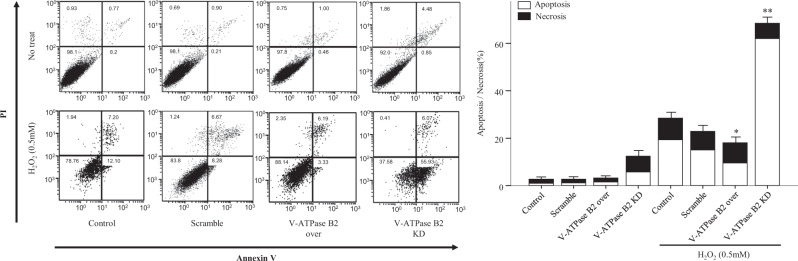
Effect of knockdown or overexpression of V-ATPase B2 on alveolar epithelial (A549) cell death. Stable V-ATPase B2-overexpressing or V-ATPase B2-knockdown cells were treated with H_2_O_2_ (0.5 mM) for 24 h in serum-free medium. Apoptosis was defined by Annexin V^+^/both PI^+^ and PI^−^ staining. Necrosis was defined by PI^+^/both Annexin V^+^ and Annexin V^−^ staining. The data are expressed as the mean ± standard error of the mean (*n* = 4). *p < 0.05 versus apoptosis or necrosis of the H_2_O_2_-treated control. **p < 0.05 versus apoptosis or necrosis of the H_2_O_2_-treated scramble.

Excessive oxidative stress-induced alveolar epithelial cell (AEC) damage is a principal mechanism of the development and progression of chronic lung diseases, including chronic obstructive pulmonary disease, asthma, and lung fibrosis. Because excessive oxidative stress causes AEC death by apoptosis or necrosis, we investigated the role of V-ATPase B2 in oxidative stress-induced cellular damage. We constructed A549 cells stably expressing FLAG-tagged human V-ATPase B2 and found that overexpressed V-ATPase B2 protein localized to lysosomes (Supplementary Fig. 1c). After 24 h of exposure of cells to 0.5 mM H_2_O_2_, we assessed the proportions of apoptotic and necrotic cells by flow cytometry. V-ATPase B2 knockdown dramatically enhanced the H_2_O_2_-induced fractions of apoptotic and necrotic cells, whereas V-ATPase B2 overexpression significantly reduced both (Fig. 4). These results demonstrate that, under excessive oxidative stress, V-ATPase B2 is essential to maintain cellular survival and that overexpression of V-ATPase B2 can rescue cells from death in vitro.

### V-ATPase B2 regulates oxidative stress-induced LMP stabilization

Lysosomes are single membrane-bound cytoplasmic organelles containing numerous hydrolases responsible for a major part of the intracellular turnover of proteins and macromolecules^32^. Oxidative stress can destabilize the lysosomal membrane by causing lipid peroxidation and damaging lysosomal membranes in a process referred to as LMP^8^. Because V-ATPase B2 is localized in lysosomes, we were interested in whether V-ATPase B2 could regulate oxidative stress-induced LMP, because it has recently been discovered that apoptosis and necrosis are often associated with LMP^10^^.^ A distinctive sign of LMP is the translocation of soluble lysosomal components from the lysosomal lumen to the cytosol^8^. We investigated H_2_O_2_-triggered LMP in three types of cells, WT, V-ATPase B2-overexpressing, and V-ATPase B2-knockdown A549 cells, using two methods: assessment of the decrease in lysosomal acridine orange (AO) staining and assessment of the release of cathepsin D, the most abundant lysosomal protease, into the cytosol. AO is a lysosomotropic metachromatic dye that yields red fluorescence when it accumulates within lysosomes and green fluorescence when it diffuses into cytosol and nuclei^8^. AO-loaded cells manifest reduced red fluorescence, while green fluorescence increases after LMP^33^. Lysosomal rupture was evaluated by assessment of the percentage increase in mean green fluorescence, as quantified by flow cytometry in the FL1 channel.

**Fig. 5:**
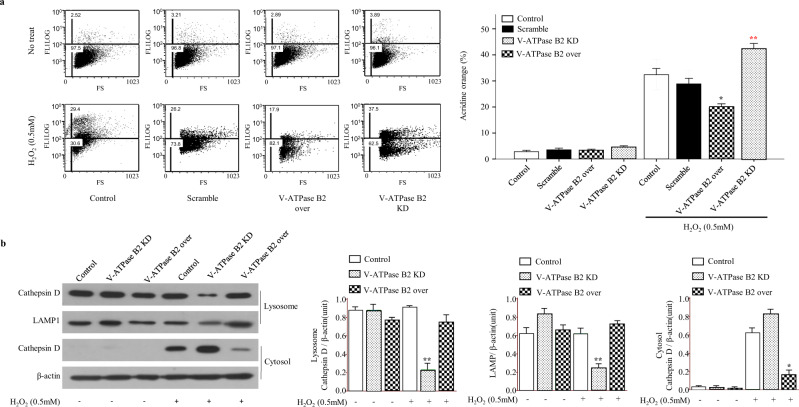
Effect of knockdown or overexpression of V-ATPase B2 in alveolar epithelial (A549) cells on H_2_O_2_-induced lysosomal membrane permeabilization (LMP). **a** Cells were preloaded with acridine orange (AO) (5 μg/mL) for 30 min in complete culture medium, rinsed, and then exposed to H_2_O_2_ (0.5 mM) for 17 h. The increase in green fluorescence due to the release of AO from ruptured lysosomes was quantified by flow cytometry in the FL1 channel. *p < 0.05, H_2_O_2_-treated control versus the H_2_O_2_-treated V-ATPase B2 overexpression groups. **p < 0.05, H_2_O_2_-treated control versus the H_2_O_2_-treated V-ATPase B2-knockdown groups. **b** Cathepsin D Western blot analysis of the lysosomal and cytosolic fractions of V-ATPase B2-overexpressing and V-ATPase B2-knockdown cells exposed to H_2_O_2_ (0.5 mM, 17 h). In immunoblot analysis, only pcDNA 3.1-Flag was used as the control. The data are expressed as the mean ± standard error of the mean (*n* = 4). *p < 0.05, H_2_O_2_-treated control versus the H_2_O_2_-treated V-ATPase B2 overexpression groups. **p < 0.05, H_2_O_2_-treated control versus the H_2_O_2_-treated V-ATPase B2 knockdown groups.

When we quantified AO fluorescence by flow cytometry in the presence of H_2_O_2_, the mean green fluorescence value was significantly higher in V-ATPase B2-knockdown cells than in scramble cells (Fig. 5a). In contrast, LMP was significantly decreased in V-ATPase B2-overexpressing cells compared to controls or V-ATPase B2-knockdown cells (Fig. 5a). We also measured cathepsin D in the cytosol of H_2_O_2_-exposed cells by Western blotting. In WT cells, cathepsin D was lost from the membrane fraction (which also contained lysosomes) and appeared in the cytosol after 60 min of exposure to H_2_O_2_. Note that, as for LMP, the lysosomal loss and concomitant cytosolic appearance of cathepsin D proteins were accelerated in V-ATPase B2-knockdown cells but were effectively prevented in V-ATPase B2-overexpressing cells (Fig. 5b). Given this surprising finding, it was important to demonstrate that LMP and cathepsin D release were effectively regulated by the V-ATPase B2 protein in response to oxidative stress.

### V-ATPase B2 overexpression enhances collagen uptake and degradation

**Fig. 6:**
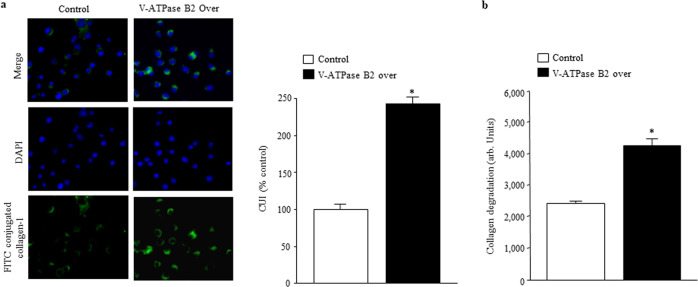
V-ATPase B2-overexpressing macrophages exhibit enhanced collagen uptake and degradation. Effects of V-ATPase B2 overexpression on **a** collagen uptake and **b** degradation by fluorescein isothiocyanate (FITC)-conjugated collagen type I in V-ATPase B2-overexpressing macrophages as measured using confocal microscopy and fluorescence spectroscopy. **a** Ingestion of collagen quantified as the collagen uptake index (CUI: number of macrophages with ingestion divided by the total number of macrophages counted). *p < 0.05 versus the control groups. **b** Cells were plated at a concentration of 50,000 cells per well in phosphate-buffered saline with 1 μg/mL FITC-conjugated type I collagen in a total volume of 100 μL. FITC-conjugated collagen (Invitrogen) is designed for enzymatic assays and is supersaturated with FITC. When collagen is cleaved, the fluorescent signal increases. After 2 h, fluorescence was quantified using a spectrofluorometer. The fluorescent signal of control wells containing only collagen was subtracted from that of wells containing macrophages and collagen. The data are expressed as the mean ± standard error of the mean (*n* = 4). *p < 0.05 versus the control groups.

The production, deposition, and remodeling of collagen, a major component of the extracellular matrix (ECM), is a dynamic process. In healthy lungs, there is a balance between the degradation and synthesis of collagen and other ECM proteins; this balance is disturbed in chronic lung diseases, including IPF^34^. Collagen in the ECM is cleaved by proteases and consequently routed to lysosomes for complete degradation^35^. Because phagocytosis of collagen is mainly executed by macrophages^36^, we used macrophages stably overexpressing V-ATPase B2 to test whether overexpression of V-ATPase B2 activates collagen uptake and degradation using an in vitro collagen phagocytosis assay (Fig. 6a). All macrophages were cultured with FITC-conjugated type I collagen, and the internalization of collagen was quantified by fluorescence microscopy. Overexpression in macrophages significantly increased collagen uptake (Fig. 6a).

Next, we were interested in whether macrophages overexpressing V-ATPase B2 exhibit altered collagen-degrading capacity. FITC-conjugated collagen molecules are supersaturated with fluorescein such that the proximity of the fluorescein molecules quenches the FITC fluorescent signal. Proteolytic degradation of collagen results in separation of the fluorescent dye and an increase in the fluorescent signal, which can then be used to quantify collagen cleavage. We therefore cultured V-ATPase B2-overexpressing or control macrophages for 80 min with FITC-conjugated type I collagen and measured the fluorescent signal to quantify collagen degradation. We found that V-ATPase B2 overexpression significantly enhanced collagen degradation in macrophages (Fig. 6b).

### Doxycycline-induced overexpression of human V-ATPase B2 in transgenic mice

Immunoblot analysis with an anti-FLAG antibody showed that the human V-ATPase B2 protein was expressed in the lungs of the V-ATPase B2 transgenic mice only after doxycycline treatment, suggesting that V-ATPase B2 expression is tightly regulated by doxycycline (Supplementary Fig. 2b). Immunofluorescence analysis revealed strong staining for V-ATPase B2 in alveolar epithelial cells following treatment with doxycycline (Supplementary Fig. 2c).

### Overexpression of V-ATPase B2 protects against bleomycin-induced lung inflammation and fibrosis

**Fig. 7:**
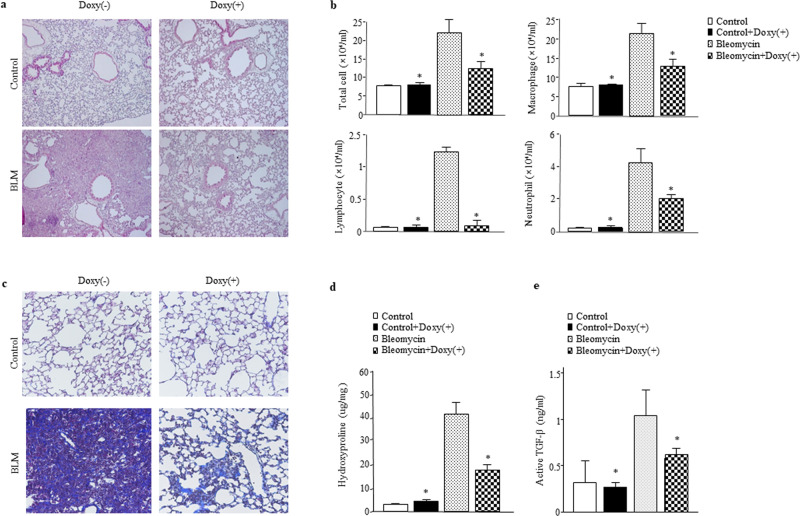
Overexpression of V-ATPase B2 protects against BLM-induced lung inflammation and fibrosis. **a** Photographs of hematoxylin and eosin staining in control and BLM-treated mouse lungs treated with doxycycline. Lung samples were collected on Day 14. Magnification: 100×. **b** Cell counts in bronchoalveolar lavage (BAL) fluid that was collected on Day 14. The cells were counted using a hemocytometer. Differences between BAL fluid cell counts were analyzed based on 500 cells stained with Diff-Quik (*n* = 8/group). *p < 0.05 versus the BLM-treated group. **c** Masson’s trichrome staining. Magnification: 100×. **d** Collagen measurements from hydroxyproline assays of control and BLM-treated mouse lungs with and without doxycycline (*n* = 8/group). **e** Active transforming growth factor (TGF)-β1 levels in control and BLM-treated mouse lungs with and without doxycycline (*n* = 8/group). Active TGF-β1 in lung lysates was quantified using enzyme-linked immunosorbent assays. *p < 0.05 versus the BLM-treated group.

To determine the role of V-ATPase B2 in the development of experimental lung injury and fibrosis, V-ATPase B2 transgenic overexpression (TO) and control littermates were challenged with intratracheal BLM (2 U/kg) at Day 0 and sacrificed at Day 14. Histological examination of the control group mice showed interstitial edema with extensive accumulation of inflammatory cells, as well as destruction of the alveolar structure (Fig. 7a). The total numbers of inflammatory cells, macrophages, neutrophils, and lymphocytes in the BAL fluid were significantly decreased in the TO group compared to the control group (Fig. 7b). To evaluate antifibrotic activity, we performed Masson’s trichrome staining and assessed the total hydroxyproline content in the lungs.

Masson’s trichrome staining revealed a decrease in collagen deposition in the lungs in the TO group compared to the control group (Fig. 7c). The lung levels of hydroxyproline were also significantly reduced in the TO group (Fig. 7d). The levels of the active form of transforming growth factor-β1 in the lungs were significantly increased following treatment with BLM but were significantly decreased in the TO group (Fig. 7e).

## Discussion

We identified a role of lysosomal V-ATPase B2 and its beneficial effect in an oxidative stress-induced cellular damage and lung fibrosis model. We observed that overexpression of V-ATPase B2 downregulates cellular apoptosis and necrosis and is associated with enhancement of lysosomal activities and stabilization of LMP against oxidative stress. We also showed that V-ATPase B2 transgenic mice exhibit attenuated BLM-induced lung inflammation and fibrosis. In addition, we observed that macrophages overexpressing V-ATPase B2 exhibit enhanced collagen uptake and degradation. This study is the first to report on the role of V-ATPase B2 and its therapeutic implication in oxidative stress-induced lung injury/fibrosis.

V-ATPase is a lysosomal core protein involved in the acidification of intracellular organelles. V-ATPases play an important role in normal physiological processes such as receptor-mediated endocytosis, intracellular membrane trafficking, prohormone processing, and protein degradation^37^. V-ATPase is a large multisubunit complex organized into two domains. The peripheral V_1_ domain is composed of eight different subunits (A–H) and functions to hydrolyze ATP^37^. Among the subunits, B2 is ubiquitously expressed and predominantly localized to intracellular compartments, but its role has not been clearly identified. Previously, we reported that V-ATPase B2 protein expression increases in TiO_2_-exposed bronchial epithelial cells^19^. Since TiO_2_ is one of the main components of air pollution and induces ROS production in the lungs^38^, we investigated whether V-ATPase B2 expression was also elevated in oxidative stress-induced human lung diseases, such as IPF, and in a BLM-induced lung injury/fibrosis model. We found that V-ATPase B2 expression was increased in all four IPF lung tissues evaluated compared to the control samples (Fig. 1a). In line with this, BLM enhanced V-ATPase B2 expression in mouse lungs (Fig. 1b). These results suggest that increased expression of V-ATPase B2 protein in the lungs occurs in response to excessive oxidative stress.

Next, we examined the role of V-ATPase B2 using stable V-ATPase B2-overexpressing and V-ATPase B2-knockdown cells. We found that the basal-state lysosomal pH was lower in cells overexpressing V-ATPase B2 than in control cells, in agreement with the enhanced lysosomal activity (Fig. 2a, b). We found that a more acidic pH, measured using LysoTracker dye, was maintained in the overexpressing cells upon H_2_O_2_ exposure (Fig. 2c–e). In addition, lysosomal proteolysis, such as degradation of BSA, was greatly increased in the overexpressing cells (Fig. 3). These results suggest that V-ATPase B2 overexpression potentiates lysosomal activity, particularly under oxidative stress.

Excessive oxidative stress can trigger lysosomal damage with the subsequent release of proteolytic enzymes into the cytosol, frequently leading to necrotic or apoptotic cell death^39^. Our results demonstrate that V-ATPase B2 overexpression significantly reduces the increase in H_2_O_2_-induced apoptotic and necrotic cell death (Fig. 4). This demonstrates that V-ATPase B2 is crucial to maintain cellular survival under excessive oxidative stress and that overexpression of V-ATPase B2 can rescue cells from death. Due to their high hydrolase content, lysosomes are potentially harmful to cells when damage occurs to the lysosomal membrane, inducing LMP^5^. LMP causes the release of cathepsins and other hydrolases from the lysosomal lumen to the cytosol, which is a critical step in lysosome-mediated apoptosis^4^. In this study, H_2_O_2_-induced LMP was monitored by various methods, including AO-loaded staining, and the translocation of cathepsin D from lysosomes to the cytosol was assessed by flow cytometry and immunoblot assays (Fig. 5a, b). We observed that cells overexpressing V-ATPase B2 dramatically reduced H_2_O_2_-induced LMP (Fig. 5a, b). Collectively, these results indicate that V-ATPase B2 plays a critical role in maintaining cellular survival and that overexpression of V-ATPase B2 has a protective activity against oxidative stress through stabilizing LMP, but its detailed mechanisms require further clarification.

Collagen is the main ECM molecule, and its excessive deposition is a characteristic feature of IPF pathology^40^. The collagen content in the ECM is strictly regulated by the balance between collagen production and degradation, and tissue fibrosis occurs when production exceeds degradation^41^. To date, little is known about the pathways responsible for collagen removal; in the ECM, collagen fibers are cleaved and denatured by matrix metalloproteinases and further degraded by gelatinases^42–44^. The intracellular pathway of collagen degradation involves phagocytosis and lysosomal degradation of collagen by cathepsins^45–47^. To determine whether increased lysosomal activity by V-ATPase B2 regulates collagen turnover, collagen uptake and degradation assays were performed in macrophages overexpressing V-ATPase B2. Interestingly, the macrophages showed both significantly enhanced uptake and degradation (Fig. 6a, b). These results provide evidence that lysosomes play an important role in collagen turnover and that enhancing lysosomal activity by V-ATPase B2 overexpression can downregulate experimental lung fibrosis.

Next, we investigated the role of V-ATPase B2 in BLM-induced experimental lung injury and fibrosis using V-ATPase B2 transgenic mice, which showed protection from BLM-induced lung damage and fibrosis (Fig. 7). Taken together, our results demonstrate that V-ATPase B2 overexpression has a protective function against oxidative stress and might be a therapeutic target for lung injury and fibrosis.

The present study had some limitations. First, we did not evaluate whether V-ATPase B2 overexpression can regulate endoplasmic reticulum (ER) stress since increased V-ATPase activity regulates ER stress and cell death^39,48^. We also did not investigate the effect of V-ATPase B2 on the autophagic process, one of the main cell death mechanisms for maintaining cellular homeostasis^49^. Second, regarding the antifibrotic activities of V-ATPase B2 overexpression, we did not evaluate the role of V-ATPase B2 or V-ATPase activity in the precise mechanisms of fibrogenesis, such as the epithelial-to-mesenchymal transition pathway and TGF-b1 signaling. In addition, although other lysosomal subunits also play a role in maintaining lysosomal activities, we have not investigated the interaction between V-ATPase B2 and other V-ATPase subunits. These knowledge gaps need to be addressed in the near future. However, to our knowledge, these data are the first to demonstrate the role of V-ATPase subunits and show the beneficial effect of V-ATPase B2 overexpression on cell death in vitro and in vivo.

In summary, we elucidated the role of V-ATPase B2 in regulating lysosomal activity and found that overexpression of V-ATPase B2 protected against BLM-induced experimental lung injury and fibrosis. The mechanisms of these beneficial effects were stabilization of LMP and enhancement of collagen uptake and degradation. Further studies on V-ATPase B2 will contribute to our understanding of the mechanism of fibrosis and potentially lead to the development of V-ATPase B2 enhancers or mimetics for treatment of lung injury and fibrosis, including IPF.

The English in this document has been checked by at least two professional editors, both native speakers of English. For a certificate, please see: http://www.textcheck.com/certificate/u8oUXf.

## Supplementary information

Supplementary information

## References

[1] Handy DE, Loscalzo J (2012). Redox regulation of mitochondrial function. Antioxid. Redox Signal..

[2] Selman M, King TE, Pardo A (2001). Idiopathic pulmonary fibrosis: prevailing and evolving hypotheses about its pathogenesis and implications for therapy. Ann. Intern. Med..

[3] Saftig P, Klumperman J (2009). Lysosome biogenesis and lysosomal membrane proteins: trafficking meets function. Nat. Rev. Mol. Cell Biol..

[4] Villamil Giraldo AM, Appelqvist H, Ederth T, Öllinger K (2014). Lysosomotropic agents: impact on lysosomal membrane permeabilization and cell death. Biochem. Soc. Trans..

[5] Liu WJ (2015). Urinary proteins induce lysosomal membrane permeabilization and lysosomal dysfunction in renal tubular epithelial cells. Am. J. Physiol. Ren. Physiol..

[6] Repnik U, Stoka V, Turk V, Turk B (2012). Lysosomes and lysosomal cathepsins in cell death. Biochim. Biophys. Acta.

[7] Appelqvist H, Wäster P, Kågedal K, Öllinger K (2013). The lysosome: from waste bag to potential therapeutic target. J. Mol. Cell Biol..

[8] Boya P, Kroemer G (2008). Lysosomal membrane permeabilization in cell death. Oncogene.

[9] Repnik U, Česen MH, Turk B (2014). Lysosomal membrane permeabilization in cell death: concepts and challenges. Mitochondrion.

[10] Johansson A-C (2010). Regulation of apoptosis-associated lysosomal membrane permeabilization. Apoptosis.

[11] Stevens TH, Forgac M (1997). Structure, function and regulation of the vacuolar (H+)-ATPase. Annu. Rev. Cell. Dev. Biol..

[12] van Weert AW, Dunn KW, Geuze HJ, Maxfield FR, Stoorvogel W (1995). Transport from late endosomes to lysosomes, but not sorting of integral membrane proteins in endosomes, depends on the vacuolar proton pump. J. Cell Biol..

[13] Strisciuglio P, Creek KE, Sly WS (1984). Complementation, cross correction, and drug correction studies of combined β-galactosidase neuraminidase deficiency in human fibroblasts. Pediatr. Res..

[14] Loss D, DiVincenzo DP, Grinstein G (1992). Suppression of tunneling by interference in half-integer-spin particles. Phys. Rev. Lett..

[15] Ohsumi Y, Anraku Y (1981). Active transport of basic amino acids driven by a proton motive force in vacuolar membrane vesicles of Saccharomyces cerevisiae. J. Biol. Chem..

[16] Ohsumi Y, Anraku Y (1983). Calcium transport driven by a proton motive force in vacuolar membrane vesicles of Saccharomyces cerevisiae. J. Biol. Chem..

[17] Tanida H, Miura A, Tanaka T, Yoshimoto T (1995). Behavioral response to humans in individually handled weanling pigs. Appl. Anim. Behav. Sci..

[18] Miller, G. J. *Multivalent Carbohydrate Ligands: Synthesis and Applications*. (The University of Manchester, United Kingdom, 2004).

[19] Cha M-H (2007). Proteomic identification of macrophage migration-inhibitory factor upon exposure to TiO_2_ particles. Mol. Cell. Proteom..

[20] Baek AR (2016). Apolipoprotein A1 inhibits TGF-β1–induced epithelial-to-mesenchymal transition of alveolar epithelial. Cells Tuberc. Respir. Dis..

[21] Ribou A, Vigo J, Salmon J (2002). C-SNARF-1 as a fluorescent probe for pH measurements in living cells: two-wavelength-ratio method versus whole-spectral-resolution method. J. Chem. Educ..

[22] Ohoka N, Yoshii S, Hattori T, Onozaki K, Hayashi H (2005). TRB3, a novel ER stress‐inducible gene, is induced via ATF4–CHOP pathway and is involved in cell death. EMBO J..

[23] Trivedi NS, Wang HW, Nieminen AL, Oleinick NL, Izatt JA (2000). Quantitative analysis of Pc 4 localization in mouse lymphoma (LY‐R) cells via double‐label confocal fluorescence microscopy. Photochem. Photobiol..

[24] Lin Y, Epstein DL, Liton PB (2010). Intralysosomal iron induces lysosomal membrane permeabilization and cathepsin D–mediated cell death in trabecular meshwork cells exposed to oxidative stress. Invest. Ophthalmol. Vis. Sci..

[25] Oberle C (2010). Lysosomal membrane permeabilization and cathepsin release is a Bax/Bak-dependent, amplifying event of apoptosis in fibroblasts and monocytes. Cell Death Differ..

[26] Szoka F, Papahadjopoulos D (1978). Procedure for preparation of liposomes with large internal aqueous space and high capture by reverse-phase evaporation. Proc. Natl Acad. Sci. USA.

[27] Corazzi L, Pistolesi R, Arienti G (1991). The fusion of liposomes to rat brain microsomal membranes regulates phosphatidylserine synthesis. J. Neurochem..

[28] Atabai K (2009). Mfge8 diminishes the severity of tissue fibrosis in mice by binding and targeting collagen for uptake by macrophages. J. Clin. Investig..

[29] Lee E (2013). Overexpression of apolipoprotein A1 in the lung abrogates fibrosis in experimental silicosis. PLoS One.

[30] Kim MS (2019). IL-37 attenuates lung fibrosis by inducing autophagy and regulating TGF-β1 production in mice. J. Immunol..

[31] Mindell JA (2012). Lysosomal acidification mechanisms. Annu. Rev. Physiol..

[32] Turk B, Turk V (2009). Lysosomes as “suicide bags” in cell death: myth or reality?. J. Biol. Chem..

[33] Antunes F, Cadenas E, Brunk UT (2001). Apoptosis induced by exposure to a low steady-state concentration of H_2_O_2_ is a consequence of lysosomal rupture. Biochem. J..

[34] Hynes RO (2009). The extracellular matrix: not just pretty fibrils. Science.

[35] Madsen DH (2007). Extracellular collagenases and the endocytic receptor, urokinase plasminogen activator receptor-associated protein/Endo180, cooperate in fibroblast-mediated collagen degradation. J. Biol. Chem..

[36] Lucattelli M (2003). Collagen phagocytosis by lung alveolar macrophages in animal models of emphysema. Eur. Respir. J..

[37] Toei M, Saum R, Forgac M (2010). Regulation and isoform function of the V-ATPases. Biochemistry.

[38] Wang J, Fan Y (2014). Lung injury induced by TiO_2_ nanoparticles depends on their structural features: size, shape, crystal phases, and surface coating. Int. J. Mol. Sci..

[39] Guicciardi ME, Leist M, Gores GJ (2004). Lysosomes in cell death. Oncogene.

[40] Fulmer JD (1980). Collagen concentration and rates of synthesis in idiopathic pulmonary fibrosis. Am. Rev. Respir. Dis..

[41] McKleroy W, Lee T-H, Atabai K (2013). Always cleave up your mess: targeting collagen degradation to treat tissue fibrosis. Am. J. Physiol. Lung Cell. Mol. Physiol..

[42] Egeblad M, Werb Z (2002). New functions for the matrix metalloproteinases in cancer progression. Nat. Rev. Cancer.

[43] Lee H, Overall CM, McCulloch CA, Sodek J (2006). A critical role for the membrane-type 1 matrix metalloproteinase in collagen phagocytosis. Mol. Biol. Cell.

[44] Visse R, Nagase H (2003). Matrix metalloproteinases and tissue inhibitors of metalloproteinases: structure, function, and biochemistry. Circ. Res..

[45] Everts V, van der Zee E, Creemers L, Beertsen W (1996). Phagocytosis and intracellular digestion of collagen, its role in turnover and remodelling. Histochem. J..

[46] Dyer RF, Peppler RD (1977). Intracellular collagen in the nonpregnant and IUD‐containing rat uterus. Anat. Rec..

[47] Melcher A, Chan J (1981). Phagocytosis and digestion of collagen by gingival fibroblasts in vivo: a study of serial sections. J. Ultrastruct. Res..

[48] Lee GH (2011). Enhanced lysosomal activity is involved in Bax inhibitor-1-induced regulation of the endoplasmic reticulum (ER) stress response and cell death against ER stress: involvement of vacuolar H+-ATPase (V-ATPase). J. Biol. Chem..

[49] Denton D, Kumar S (2019). Autophagy-dependent cell death. Cell Death Differ..

