# Supplementary material for: Overexpression of V-ATPase B2 attenuates lung injury/fibrosis by stabilizing lysosomal membrane permeabilization and increasing collagen degradation
Source: Exp Mol Med. 2022 May 27;54(5):662–72. doi: 10.1038/s12276-022-00776-2 (PMC9166714; doi:10.1038/s12276-022-00776-2)
Supplement: Supplementary file 1 — Supplementary information [file 12276_2022_776_MOESM1_ESM.pdf]

Overexpression of V-ATPase B2 attenuates lung injury/fibrosis by stabilizing lysosomal membrane permeabilization and increasing collagen degradation

Jong-Uk Lee[1], Jisu Hong[1], Hyesun Shin[2], Chnag-Beom Ryu[1, **], Sung-Woo Park[1, **], Sung Hwan Jeong[3]

[1]Department of Internal Medicine, Soonchunhyang University Bucheon Hospital, 14584 Gyeonggi-Do, South Korea. [2]Department of Interdisciplinary Program in Biomedical Science Major, Soonchunhyang University Bucheon Hospital, 14584 Gyeonggi-Do, South Korea. [3]Department of Allergy, Pulmonary and Critical Care Medicine, Gachon University, Gil Medical Center, Incheon, South Korea.

Supplementary information

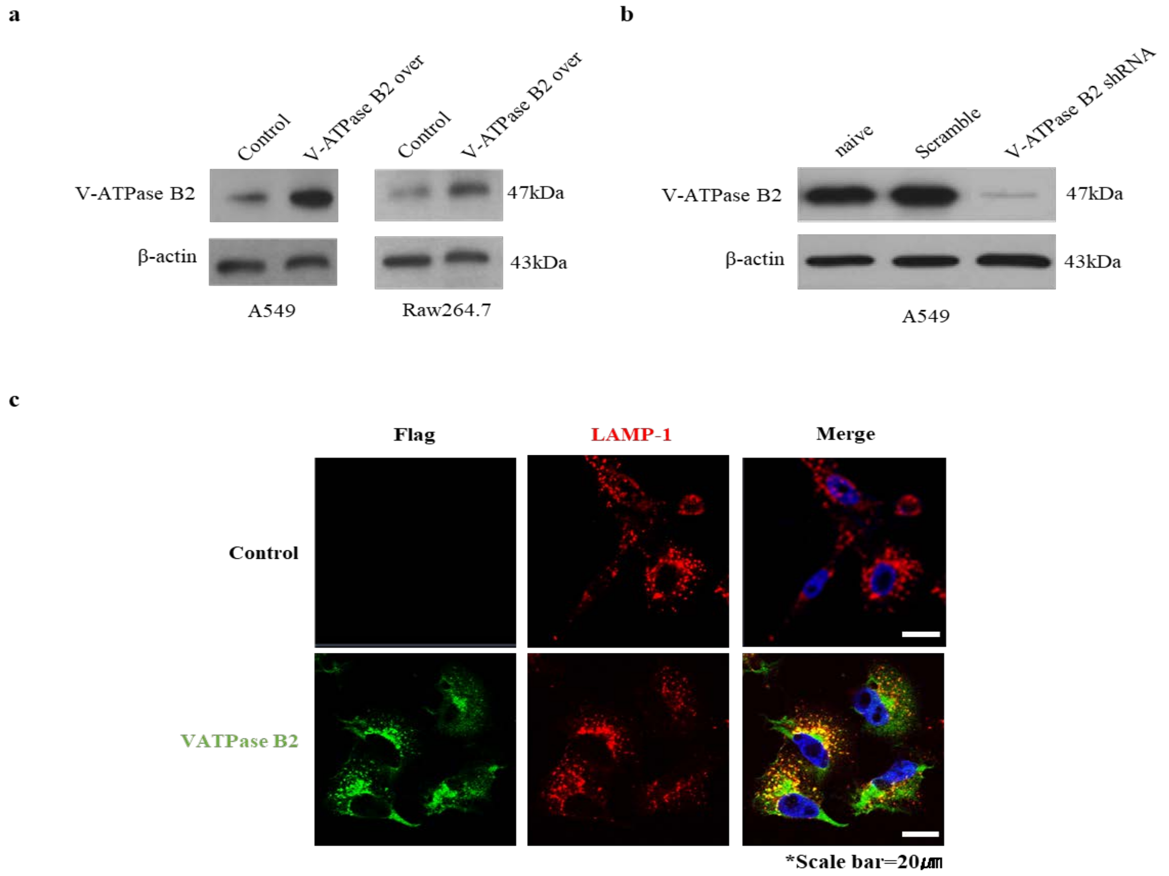

Supplementary Fig. 1. Generation of **a.** pcDNA V-ATPase B2-FLAG overexpressing and **b.** V-ATPase B2 knockdown stable cells. **c.** Representative double immunofluorescence-stained images of V-ATPase B2 overexpressing A549 cells. FLAG and LAMP-1 were stained using fluorescein isothiocyanate-conjugated antibodies (green) and R-phycoerythrin (red), respectively. Magnification: 400×.

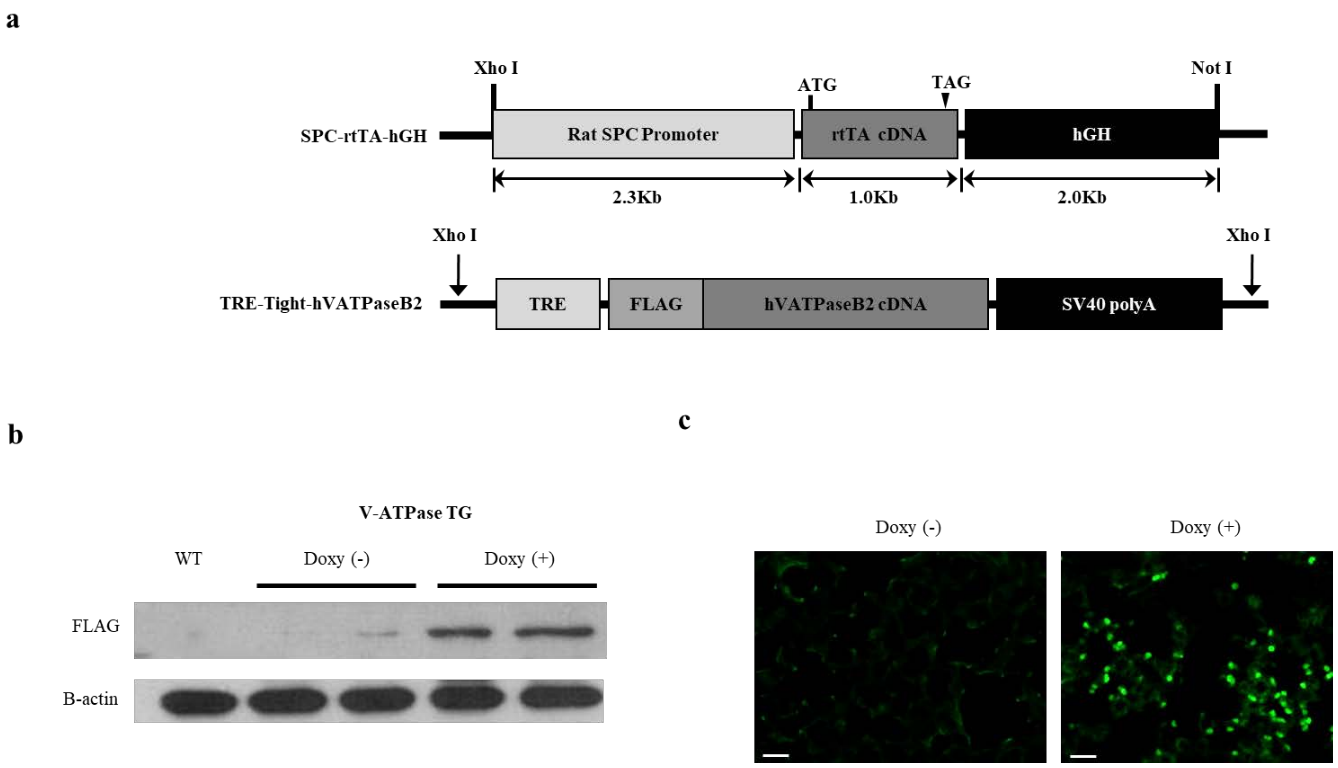

Supplementary Fig. 2. Generation of V-ATPase B2 transgenic mice.

Overexpression of human V-ATPase B2 was induced using doxycycline-containing drinking water (Doxy+). Control mice were offered normal water (Doxy–). **a.** Constructs for generation of doxycycline inducible alveolar epithelial cell-specific human V-ATPase B2-expressing transgenic mice. **b.** Human V-ATPase B2 protein expression was significantly greater in the lungs of doxycycline-treated transgenic mice compared to those of wild-type or transgenic mice not treated with doxycycline (n = 6 mice/group). *$p < 0.01$ compared to wild-type (WT) or Doxy (–) mice.

c. Immunofluorescence analysis showing that human V-ATPase B2 was strongly expressed in the alveolar epithelium in the doxycycline-treated transgenic mice. Scale bar = 10 μm.
